# Eosinophilic gastroenteritis in a young girl – long term remission under Montelukast

**DOI:** 10.1186/1471-230X-5-24

**Published:** 2005-07-18

**Authors:** Ivo Quack, Lorenz Sellin, Nikolaus J Buchner, Dirk Theegarten, Lars C Rump, Bernhard F Henning

**Affiliations:** 1 Department of Internal Medicine, Gastroenterology Unit, Marienhospital, Ruhr University, Herne, Germany; 2 Department of Pathology, Ruhr-University, Bochum, Germany

## Abstract

**Background:**

Eosinophilic gastrointestinal disorders are an emerging disease entity characterized by eosinophilic infiltration of the intestinal wall. Oral steroids can be still considered as first line treatment. Unfortunately relapses are quite common. Usually long term low-dose prednisone or immunosuppressive therapy is required, which is especially problematic in young patients. Thus a reliable steroid sparing agent with low side effects suitable for long term use is needed. There are strong hints to a similar pathophysiology of eosinophilic gastrointestinal disorders to that of asthma. Indeed leukotriene D4 plays an important role in the recruitment of eosinophils into the intestinal tissue causing damage. This patho-mechanism provides the rationale for the treatment with a leukotriene D4 receptor antagonist. Recently there have been first reports about successful short term use of Montelukast in eosinophilic gastrointestinal disorders.

**Case presentation:**

We report the case of a 17 year old girl with a long history of severe abdominal complaints leading to several hospitalizations in the past. Mimicking the picture of an intestinal tuberculosis she received an anti mycobacterial treatment without any success. Marked eosinophilia in blood, ascites and tissue samples of the intestinal tract finally lead to the diagnosis eosinophilic gastroenteritis. Tapering off prednisone caused another severe episode of abdominal pain. At that point leukotriene antagonist Montelukast was started at a dose of 10 mg once daily. Steroids could be tapered off completely within six weeks. The patient has been free of symptoms for over two years by now. Routine examinations, blood tests and endoscopy have rendered regular results. So far no side effects were noted.

**Conclusion:**

Here report about successful long term remission of eosinophilic gastroenteritis under Montelukast. Further randomized control trials are required to asses the full benefits of Montelukast therapy in the whole spectrum of eosinophilic gastrointestinal disorders.

## Background

Eosinophilic gastroenteritis is a rare disease characterized by eosinophilic infiltration of the intestinal wall. It may mimic peptic ulcer, intestinal obstruction, gastroenteritis, irritable bowel syndrome, and inflammatory bowel disease. Long-term personal history of digestive symptoms and the course of the disease with relapses and remissions is the key for the disease to be suspected. Endoscopy, CT scan and sonographic studies may provide important indirect signs of the disease. But only in combination with histological examination the diagnosis can be achieved.

Primary eosinophilic gastrointestinal disorders (EGID) selectively affect the gastrointestinal tract with eosinophil rich inflammation in the absence of known causes for eosinophilia[1]. The natural history of EGIDs has not been well documented, however these diseases are often chronic waxing and waning disorders. The clinical presentation depends on the gastrointestinal division affected and the wall layers infiltrated.

EGIDs are primarily polygenic allergic disorders involving mechanisms that fall between IgE-mediated food allergy and cellular-mediated hypersensitivity disorders[1]. Steroids are still the main therapy for cases in which diet restriction is not feasible or has failed. The recent research suggests a similar pathophysiology to asthma[2]. Cysteinyl leukotrienes have potent chemo attractant properties for eosinophils. Together with interleukin 3 and 5 they play a major role in the recruitment of eosinophils into the tissue causing damage[3]. Disrupting this vicious circle may provide the rationale for treating patients with eosinophilic gastroenteritis with leukotriene receptor antagonists (LTRA).

Here we report a case of young women with a long history of eosinophilic gastroenteritis. Under therapy with the LTRA Montelukast she has been completely free of symptoms for over two years by now. Routine physical examinations and blood tests have rendered regular results.

## Case report

A 17-year-old girl was admitted to the local gynaecology unit with recurrent abdominal cramps, nausea and vomiting. She had a history of repeated episodes of abdominal pain in the past. The symptoms had begun some years earlier, causing several hospitalizations and two laparoscopic interventions.

Medical history revealed allergic coryza and neurodermatitis, but no food allergy. No other diseases were reported.

Laboratory rendered a high white blood cell count (WBC) 13.300 × 10^9^/liter with an eosinophil count of 58% and CRP 5.2 mg/l. Laparoscopy showed a large amount of ascites and swollen intraabdominal lymph nodes. Thus, screening for tuberculosis was initiated. A chest x-ray showed no signs of pulmonary disease, Mantoux test and all cultures remained negative. However, one pcr from ascites was positive and the diagnosis of intestinal tuberculosis was established. The patient was started on rifampicin, isoniazid, ethambutol and pyrazinamide. Despite treatment symptoms persisted for the following six month.

**Figure 1:**
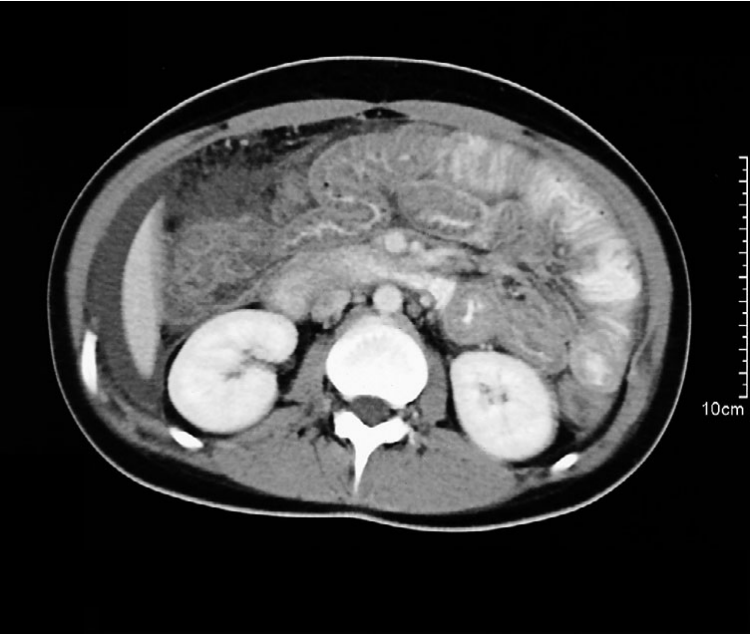
Contrast-enhanced spiral CT revealing ascites and concentric thickening of colon and ileum.

**Figure 2:**
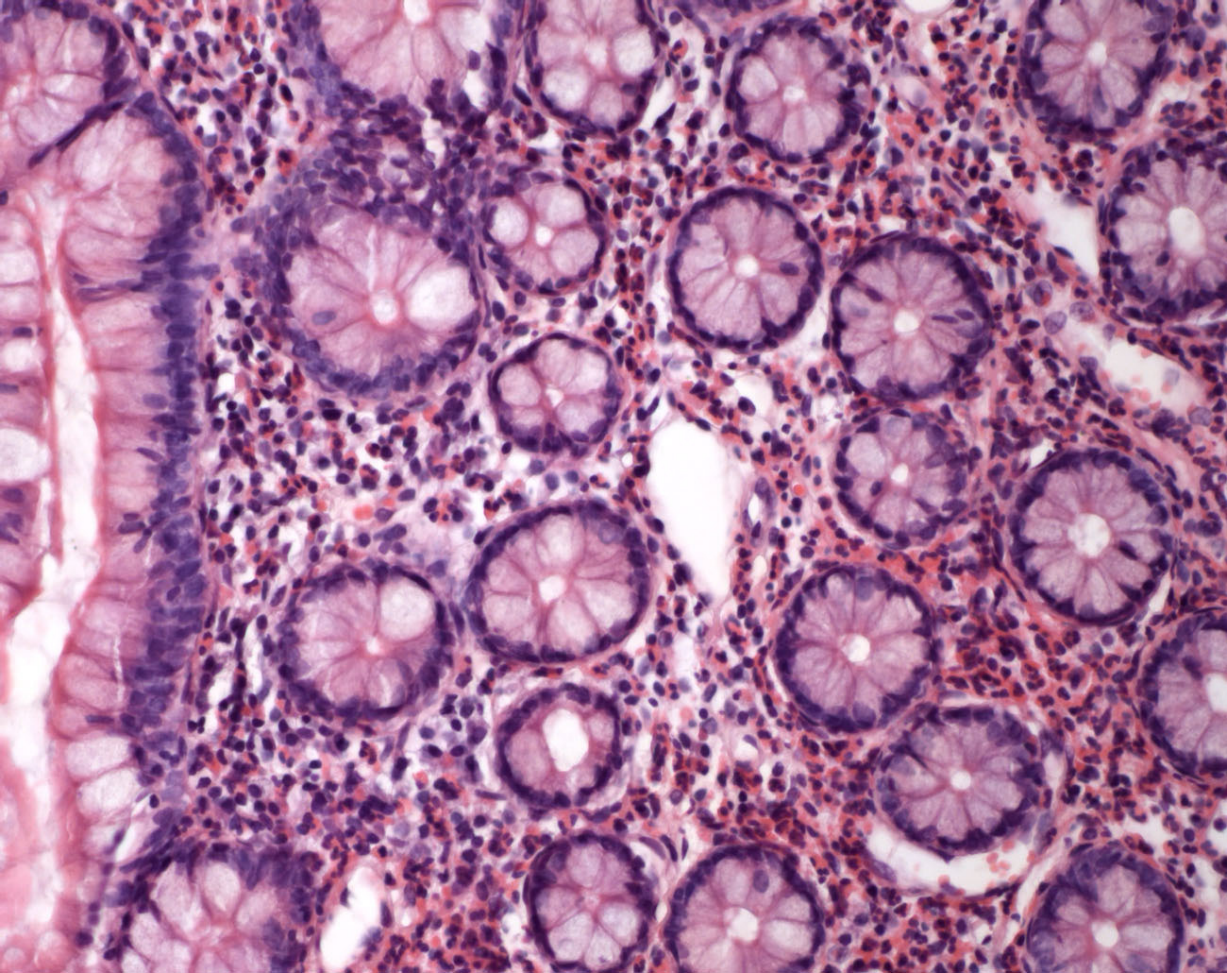
Endoscopic biopsy of ileum showing distinct eosinophilic infiltration (haematoxylin and eosin ×100).

On admission the patient was still under therapy with rifampicin and isoniazid. Physical examination demonstrated diffuse abdominal tenderness without peritoneal signs. The WBC was 13100 × 10^9^/liter with an eosinophilic count of 53 %, total IgE 1870 kU/l and CRP 32 mg/l. Clinical and laboratory indices for autoimmune disease were absent. Abdominal ultrasound and computed tomography showed ubiquitous ascites and thickening of the wall of the lower oesophagus, stomach and bowel (Fig. 1). The patient developed severe abdominal pain, only controlled by opoid analgesia. An iliac crest biopsy showed an eosinophilic count over 40 percent but no signs of leukaemia. Again the ascitic fluid rendered a high eosinophilic count. Blood tests as well as stool cultures and microscopy for ova and parasites were negative. Endoscopy of the upper intestinum and the colon showed no abnormalities of the intestinal mucosa. Remarkably biopsies from oesophagus, stomach, ileum and colon yielded distinct eosinophilic infiltrations reaching the lamina propria which finally lead to the diagnosis of a primary eosinophilic gastrointestinal disorder (Fig. 2).

Thus, this patient fulfilled all diagnostic criteria for eosinophilic gastroenteritis. Since active tuberculosis was ruled out we stopped anti-mycobacterial treatment.

The patient was switched to 40 mg prednisone per day. Symptoms were alleviated quickly in the beginning. The patient was set on a taper. Reaching a dose of 10 mg a day symptoms started again. Therefore the dose was doubled and Montelukast 10 mg/d was started. The symptoms resolved within days and the prednisone could be tapered within six weeks. Currently, the patient remains completely off steroids. She has been symptom free for the last 24 months under Montelukast. Regular physical examinations and blood tests have shown normal results.

## Discussion

EGIDs are primarily polygenic allergic disorders involving mechanisms that fall between IgE-mediated food allergy and cellular-mediated hypersensitivity disorders. They selectively affect the gastrointestinal tract with eosinophil rich inflammation in the absence of known causes for eosinophilia[1]. The clinical presentation depends on the gastrointestinal division affected and the wall layers infiltrated. Eosinophils have been shown to be integral members of the gastrointestinal mucosal immune system, but their presence in deeper layers is almost always pathologic[4]. One of their principal functions is to defend against multicellular helminthic parasites. Many of the secreted proteins are potent helminthotoxins. Other products affect smooth muscle contraction, vascular permeability and the release of other mediators from mast cells and basophiles[3].

Eosinophilic gastroenteritis is still a quite rare disease, but EGIDs are currently being recognized more frequently[5,6]. Patients with EGIDs present with a variety of clinical problems, most commonly failure to thrive, abdominal pain, irritability, gastric dysmotility, vomiting, diarrhoea and dysphagia[7]. Thus the variety of non-specific common gastrointestinal symptoms und laboratory findings explains why the correct diagnosis is completely dependent on the microscopic examination of the biopsy samples. The clinical presentation depends on the division affected and the wall layers infiltrated. Patients with primarily mucosal disease tend to present with non-specific gastrointestinal symptoms while those with serosal disease can present with severe abdominal pain and ascites[8].

Our patient suffered from recurring severe non-specific abdominal pains for about five years, causing several hospital stays and two laparoscopic interventions. Approximately one year before admission to our hospital the patient developed ascites. Eosinophilic ascites is only observed in 10 % of cases. It indicates the involvement of the tunica serosa and is typical in women of childbearing age[9]. Laparoscopy showed a swollen intestinal tract and lymph nodes resembling the aspect of intestinal tuberculosis. Thus ascites was screened for mycobacteria. One pcr screening turned out positive. Microscopy and repeated cultures from ascites, sputum and biopsy of intestinal lymph nodes remained negative for mycobacteria at all times. Lacking another coherent differential diagnosis anti mycobacterial treatment was started. Despite therapy for six month ascites and abdominal complaints remained nearly unchanged. On admission no signs of active tuberculosis were found. Skin test, chest x-ray, double pcr screening as well as cultures from sputum and ascites were negative. These findings raise the question if the tuberculosis has been treated successfully or the first pcr result has been false positive. Regarding the persisting symptoms the latter seems more probable. Furthermore international guidelines do not accept the single use of nucleic acid amplification technique to establish the diagnosis tuberculosis[10].

The patient now presented again with same complaints which strongly suggested an eosinophilic gastrointestinal disorder. Retrospectively eosinophilia was present already at the last hospital stay which argues for EG then. Exclusion of other causes of eosinophilia, summarized in Klein's criteria[11] and histopathological confirmation of eosinophilic infiltration of the GI tract supported our initial diagnosis.

The diagnosis hypereosinophilic syndrome (HES) should always be considered in patients with EGIDs[12]. But here was no evidence of extraintestinal organ manifestation (e.g. cardiorespiratory system or skin) in our patient. Bone marrow analysis showed no signs of myodysplasia. Markers like vitamin B 12 and serum mast cell tryptase were normal[13]. The FIP1L1-PDGFRA fusion event, which is found in about half of the HES patients, was not present. It is generated by a chromosomal deletion and indicates that these cases are hematopoetic malignancies[14]. Taken together HES seemed to be very unlikely.

EG is an uncommon disease and, as such, no prospective treatment studies are available. In literature, case reports and small case series have reported positive impact from a variety of agents. Besides corticosteroids, mast cell stabilizers, antihistamins, leukotriene antagonists, octreotide as well as restriction diets and resection of stenosed intestinal segments (reviewed in [1]). Based on this data, systemic steroids still can be considered first choice therapy in non obstructive disease. Unfortunately relapses are common. These patients require long term low-dose prednisone or immunosuppressive therapy, which is problematic especially in young patients. Cysteinyl leukotrienes, along with cytokines interleukin 3 and 5 and granulocyte macrophage-colony stimulating factor seem to play a crucial role in the recruitment of eosinophils into the tissue causing damage. The leukotriene antagonist (LTRA) Montelukast blocking the leukotriene receptor Cys-LT1 could be the rationale to treat EG with this drug[15]. Anti-leukotriene drugs are considered generally safe and effective in most patients. However there have been reports about the association between LTRA and the development of Churg-Strauss-Syndrome (CSS)[16]. So clinicians need to vigilant of any development of CSS in any patient receiving these agents. Up to now no side effects were noted in our patient.

There has been one study with promising short term results in children treated with Montelukast[17]. But only two cases reporting successful control of symptoms in eosinophilic gastroenteritis in adults[18]. Schwartz and co-workers were first to report control of symptoms over twenty months[19]. One more recent study found improvement in peripheral blood but not in tissue eosinophilia or symptoms in a severe, long-standing disease[20]. The presented case now shows successful long term treatment with Montelukast over a period of two years.

## Conclusion

Eosinophilic gastroenteritis is a rare disease, but should be considered as differential diagnosis in cases of recurrent abdominal pain. Failure to diagnose this disorder often relates to reluctance to biopsy an apparently normal intestinum. Montelukast therapy seems promising to be an effective long term treatment in these patients, particularly when they are steroid dependent. So far no side effects were noted in our patient. Further randomized trials are required to asses the full benefits of Montelukast therapy in eosinophilic gastrointestinal disorders.

## Abbreviations

CSS, Churg-Strauss-syndrome; EG, eosinophilic gastroenteritis; EGID, eosinophilic gastrointestinal disorder; HES, hypereosinophilic syndrome; LTRA, leukotriene receptor antagonist; FIP1L1-PDGFRA, FIP1-like-1-platelet-derived-groth-factor-receptor-alpha fusion kinase

## Competing interests

The author(s) declare that they have no competing interests.

## Authors' contributions

IQ patient's management; design and manuscript draft

LS patient's management; help with manuscript draft and figures

NB endoscopy, reviewed history and acquired follow up data of patient

DT analysed tissue samples and provided figures

LR reviewed manuscript and literature

BH design, coordination and supervision of patient's management

All authors read and approved the final manuscript.

## Pre-publication history

The pre-publication history for this paper can be accessed here:
